# On the synergy of matrix-isolation infrared spectroscopy and vibrational configuration interaction computations

**DOI:** 10.1007/s00214-020-02682-0

**Published:** 2020-11-09

**Authors:** Dennis F. Dinu, Maren Podewitz, Hinrich Grothe, Thomas Loerting, Klaus R. Liedl

**Affiliations:** 1 Institute of General, Inorganic and Theoretical Chemistry, University of Innsbruck, Innsbruck, Austria; 2 Institute of Material Chemistry, TU Vienna, Vienna, Austria; 3 Institute of Physical Chemistry, University of Innsbruck, Innsbruck, Austria

**Keywords:** Matrix-isolation spectroscopy, Vibrational self-consistent field, Vibrational configuration interaction, Molecular vibration, Infrared spectroscopy

## Abstract

The key feature of matrix-isolation infrared (MI-IR) spectroscopy is the isolation of single guest molecules in a host system at cryogenic conditions. The matrix mostly hinders rotation of the guest molecule, providing access to pure vibrational features. Vibrational self-consistent field (VSCF) and configuration interaction computations (VCI) on ab initio multimode potential energy surfaces (PES) give rise to anharmonic vibrational spectra. In a single-sourced combination of these experimental and computational approaches, we have established an iterative spectroscopic characterization procedure. The present article reviews the scope of this procedure by highlighting the strengths and limitations based on the examples of water, carbon dioxide, methane, methanol, and fluoroethane. An assessment of setups for the construction of the multimode PES on the example of methanol demonstrates that CCSD(T)-F12 level of theory is preferable to compute (a) accurate vibrational frequencies and (b) equilibrium or vibrationally averaged structural parameters. Our procedure has allowed us to uniquely assign unknown or disputed bands and enabled us to clarify problematic spectral regions that are crowded with combination bands and overtones. Besides spectroscopic assignment, the excellent agreement between theory and experiment paves the way to tackle questions of rather fundamental nature as to whether or not matrix effects are systematic, and it shows the limits of conventional notations used by spectroscopists.

**Electronic supplementary material:**

The online version of this article (10.1007/s00214-020-02682-0) contains supplementary material, which is available to authorized users.

## Introduction

Ever since the matrix-isolation (MI) technique has been established for IR spectroscopy [1], it has played an increasingly important role in the characterization of molecular vibration and has been closely linked to computational investigations. With MI-IR spectroscopy, insights have become accessible that were previously not available through gas-, liquid- and solid-phase IR spectroscopy. In the beginnings, noble gases like argon, krypton, xenon, as well as the molecular gas nitrogen were predominantly used as host materials, because their relatively high melting points (above 60 K) allow for a comparatively uncomplicated deposition of matrix layers. Today it is possible to routinely maintain temperatures below the melting point of neon (25 K), making this noble gas increasingly available for matrix deposition. Still, experiments with neon matrices are comparatively seldom.

The inert host material is the key in each MI-IR experiment. Depositing a solid from a gas-phase mixture of inert gas and analyte at cryogenic temperatures leads to a host–guest system. Some peculiarities of MI-IR spectroscopy intricately depend on the properties of this host–guest system [2]. (1) The host exhibits weak interactions with the guest (analyte). In the spectrum, these interactions cause a shift in frequencies compared to the gas phase, the so-called *matrix shift* (typically below 4 cm^−1^ in neon matrices). (2) The host may feature different *trapping sites*, i.e., the analyte can insert into voids between host atoms, or replace one or more host atoms. Depending on the trapping site, the analyte may distort from its gas-phase structure. In the spectrum, the occupation of different trapping sites results in a splitting of bands, the so-called *matrix splitting*. (3) The matrix is solid. Hence, translational and rotational degrees of freedom are hindered or even quenched. Especially, *quenching of molecular rotation* leads to the absence of rotational–vibrational transitions in the spectrum. In some cases, such as water in noble gases [3–6], the guest molecule may still rotate in the matrix. (4) Depending on the dilution of the analyte and its tendency to form intermolecular bonds, the analyte may aggregate to form dimers, trimers, and higher oligomers. In the spectrum, additional bands occur due to *oligomerization*.

It is not straightforward to predict whether or not any of these *matrix effects* occur. A successful MI-IR study identifies all matrix effects, usually, by changing multiple parameters in the experiment, such as isotopic labeling or dilution and temperature changes. The choice of host material plays a predominant role. Although the inert gases have similar physical properties, the difference in their phase diagrams becomes crucial when approaching very low temperatures. Once the identification of matrix effects was successful, the MI-IR spectrum provides invaluable information about the vibrational structure of the trapped analyte. Howsoever, identifying matrix effects for one host–guest system does not guarantee that these matrix effects are similar in another host–guest system. In the present study, we show that matrix effects are not systematic, thus, not easily transferrable from one case to another. This investigation comprises widely studied MI-IR spectra of the monomeric species of water [3–11], carbon dioxide [12–16], methane [17–23], and methanol [24–28]. We refer to further in-depth historical accounts for water [29] as well as carbon dioxide and methane [30].

It is up to theory to conceive the information gained from an IR spectrum. Quantum mechanics provide the most successful theories to describe rotational–vibrational spectroscopy. As it is, however, impossible to analytically solve the Schrödinger equation for a molecule, it is also not for a host–guest system. Thus, a variety of approximations are necessary. First, it is suitable to neglect the host and treat the analyte *in vacuo*. When using noble gases as host materials, this approximation is sensible because the host–guest interactions are rather weak. The matrix-isolated molecule is barely distorted compared with a single molecule *in vacuo*. Furthermore, computation of the host–guest system is an expensive task: At present, this is accessible only for rather small segments using periodic boundary conditions and limited to the use of semiempirical force fields or density functional theory [31, 32]. Thus, it is sensible to focus the computational resources on the description of the analyte itself using high-level electronic structure theory.

Starting from an *in vacuo* model, it follows to separate the external and internal motion of the molecule into translation, rotation, and vibration. For each of those degrees of freedom, it is usual to introduce further approximations. At this point, various routes exist to derive a model for a molecule in motion. We refer to a variety of reviews that summarize this field of theoretical and computational chemistry [33–39]. In the present work, we consider the approaches of vibrational self-consistent field (VSCF) and vibrational configuration interaction (VCI). At the center of these computations is the accurate description of the potential energy surface (PES).

Numerous developments in quantum chemistry promise an economical and flexible, yet, accurate access to ab initio local PESs of polyatomic molecules [40–48], for which the design of global PESs would be too cumbersome and expensive. The MOLPRO software package [49] comprises a versatile and flexible computational environment for VSCF/VCI calculations. Together with state-of-the-art methods from electronic structure theory [52–54], it includes a methodologically sound implementation of multimode PES construction and VSCF/VCI computations by Rauhut et al. [36, 44, 46–48, 50, 51]. Because of the ongoing improvements of MOLPRO, it is needless to say that our calculations using the latest commercially available implementation do not necessarily reflect the final capabilities of the software package. Hence, the present paper does not aim at providing a benchmark for this particular implementation. We may refer to benchmarks by the author of the code, e.g., a juxtaposition of VSCF/VCI and perturbational calculations [55].

To demonstrate the performance of the VSCF/VCI approach when using different setups in the construction of the PES, we first provide a computational assessment. At the example of methanol, we compare our computational results to our experimental MI-IR data as well as the literature data from the experiment [28, 56–59] and other computational approaches [60–64]. Apart from this assessment, we present results from VSCF/VCI calculations as a support in the conceptualization of MI-IR spectra. Based on the molecules mentioned above (water, carbon dioxide, methane, methanol) and fluoroethane, we will provide a greater context of the insights we have gained so far. Discrepancies between theory and experiment have to be expected, as the calculations are *in vacuo*, and the experiment is not. Howsoever, we will show that the quantitative discrepancies are small enough to guarantee that the qualitative conclusions drawn from the calculations are sensible. This combined investigation with a focus on the synergy of MI-IR spectroscopy and VCI computations is the central aspect of our work.

## Methodology

### Molecular vibration from VSCF/VCI computations

**Fig. 1:**
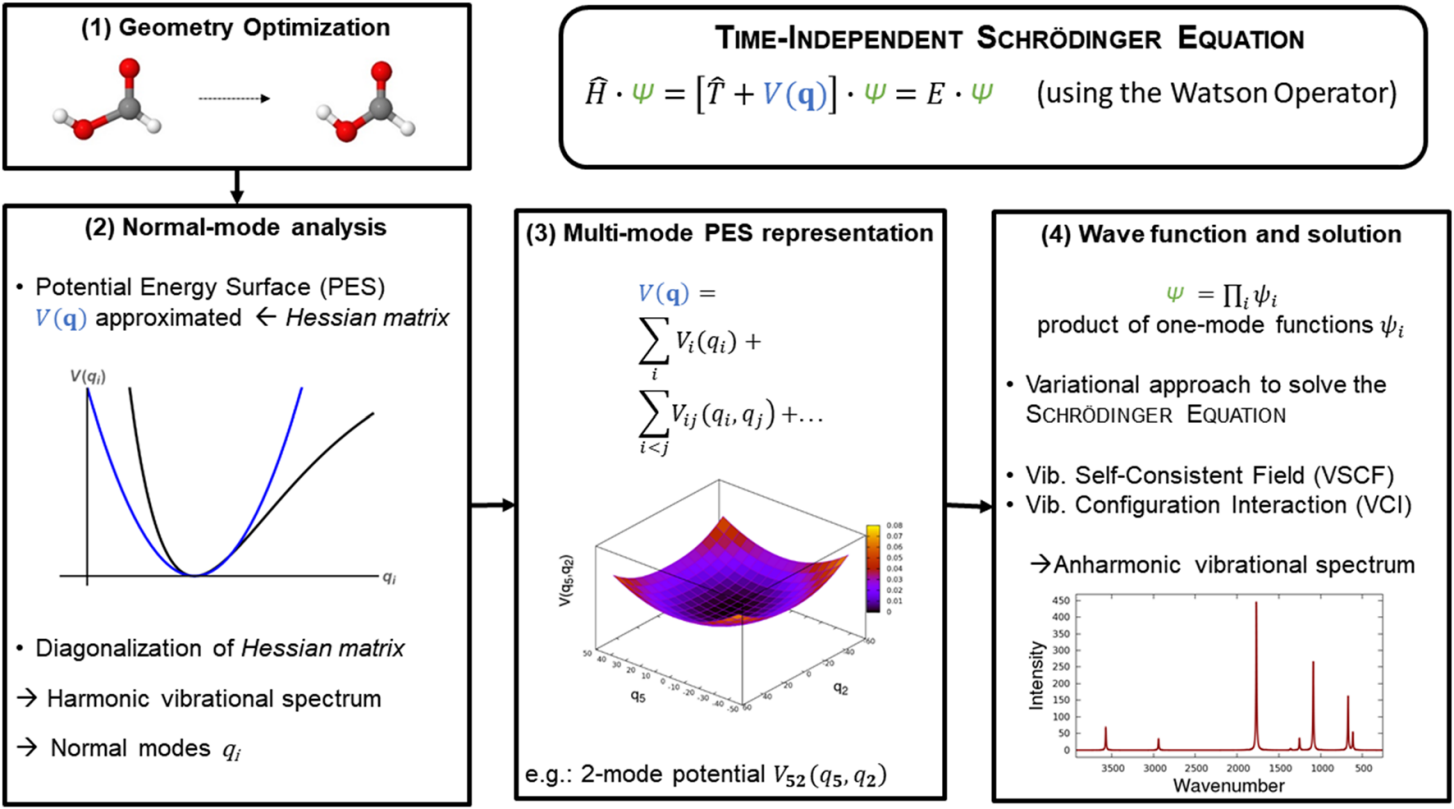
Schematic workflow of a multimode PES based VSCF/VCI calculation for solving the time-independent nuclear Schrödinger equation

Solving the time-independent nuclear Schrödinger equation provides theoretical access to states that undergo the transitions observed in IR spectroscopy. To this end, the solution of this theoretical model allows for the interpretation of IR spectra. In the particular procedure used here (Fig. 1), the Schrödinger equation using the Watson operator [65] is considered. The main ingredient of the Watson operator is the potential energy surface (PES). Bowman has pioneered to construct the PES in the Watson operator as a multimode representation [66]. The MOLPRO implementation by Rauhut [44] follows this idea. However, it additionally includes symmetry considerations, pre-screening techniques, and other numerical procedures to lower the computational costs.

For our purposes, the essential aspect of this implementation is the on-the-fly construction of the PES and its direct use for the computation of vibrational spectra. In such implementations, there is no conceptual gap between PES construction and the algorithms for solving the Schrödinger equation. Hence, the setup of these computations is comparatively simple. Although such implementations are not yet black-box approaches, they come near to what can be considered as ideally suited for large-scale combined experimental and theoretical studies of multiple molecules. Figure 1 presents the computational procedure schematically. We may crudely describe it in four steps:

1. In the beginning, a geometry optimization provides the equilibrium geometry of the molecule *in vacuo*. The PES will be expanded locally around the equilibrium geometry. It would, however, also be possible to expand the PES around a saddle-point that connects two energetically equal minima.
2. For the equilibrium geometry, a normal-mode analysis in the harmonic approximation is performed. This yields normal-mode coordinates *q*_*i*_, which define the coordinate system for the PES. In many cases of semi-rigid molecules, the use of a normal-mode coordinate system is sufficient. However, sometimes it can be of benefit to localize the normal-modes [48].
3. The multimode PES representation is a sum of potentials, with *V*_*i*_(*q*_*i*_) as one-mode potentials, *V*_*ij*_(*q*_*i*_,* q*_*j*_) as two-mode potentials, and analogous terms from higher dimensional potentials. All potentials are calculated on a grid using ab initio single-point calculations. The multimode PES is usually truncated after three-mode or four-mode potentials. Furthermore, the PES can be represented in an analytic form by the fitting of polynomials or b-splines [45, 46]. This analytic form enables transforming the PES from one isotopologue to another. The accuracy of the PES is the crucial aspect of all calculations presented here.
4. The previously constructed PES is the main ingredient for establishing the Watson operator. Using this operator, it follows to solve the time-independent Schrödinger equation. This solution is achieved in a self-consistent field (SCF) manner, constructing the wave function as a product of one-mode functions. One vibrational SCF computation is performed for each normal-mode separately. The VSCF solutions are then correlation-corrected by vibrational configuration interaction (VCI). Excitations in the VSCF reference wave function define the configurations. Formally, these excitations are obtained when a converged VSCF wave function is somewhat altered by redefining its quantum numbers. For example, in quadruple excitations, four vibrational quantum numbers are altered in the VSCF reference wave function. A VCI computation with up to quadruple excitations is termed VCI (SDTQ) or simply VCI (4). Following this recipe, a multitude of configurations is possible. As not all of the definable configurations are necessary for an accurate result, configuration-selective schemes that lower the configuration space exist [50].

In the end, the whole approach yields vibrational states that account for anharmonicities and mode-coupling. The transitions between those states are the computational counterpart to experimentally accessible IR transitions. In VCI computations, contributions of the configurations to a state provide a way to label the state energies by the conventional notations widely used in IR spectroscopy.

#### Observation of molecular vibration by MI-IR spectroscopy

On the scale of single molecules, observation of molecular vibration is directly accessible when the analyte is in the gas phase. As opposed to this, the molecules aggregate in the liquid and solid phases. Thus, the corresponding spectra are composed of broad bands that are less informative. Then again, molecules can freely rotate in the gas phase leading to rotational envelopes in the spectrum. These envelopes complicate the interpretation of gas-phase spectra. In liquid- and solid-phase spectra rotation is usually inhibited. The matrix-isolation technique combines the best of two worlds: It enables the observation of molecular vibration in non-rotating single molecules.

**Fig. 2:**
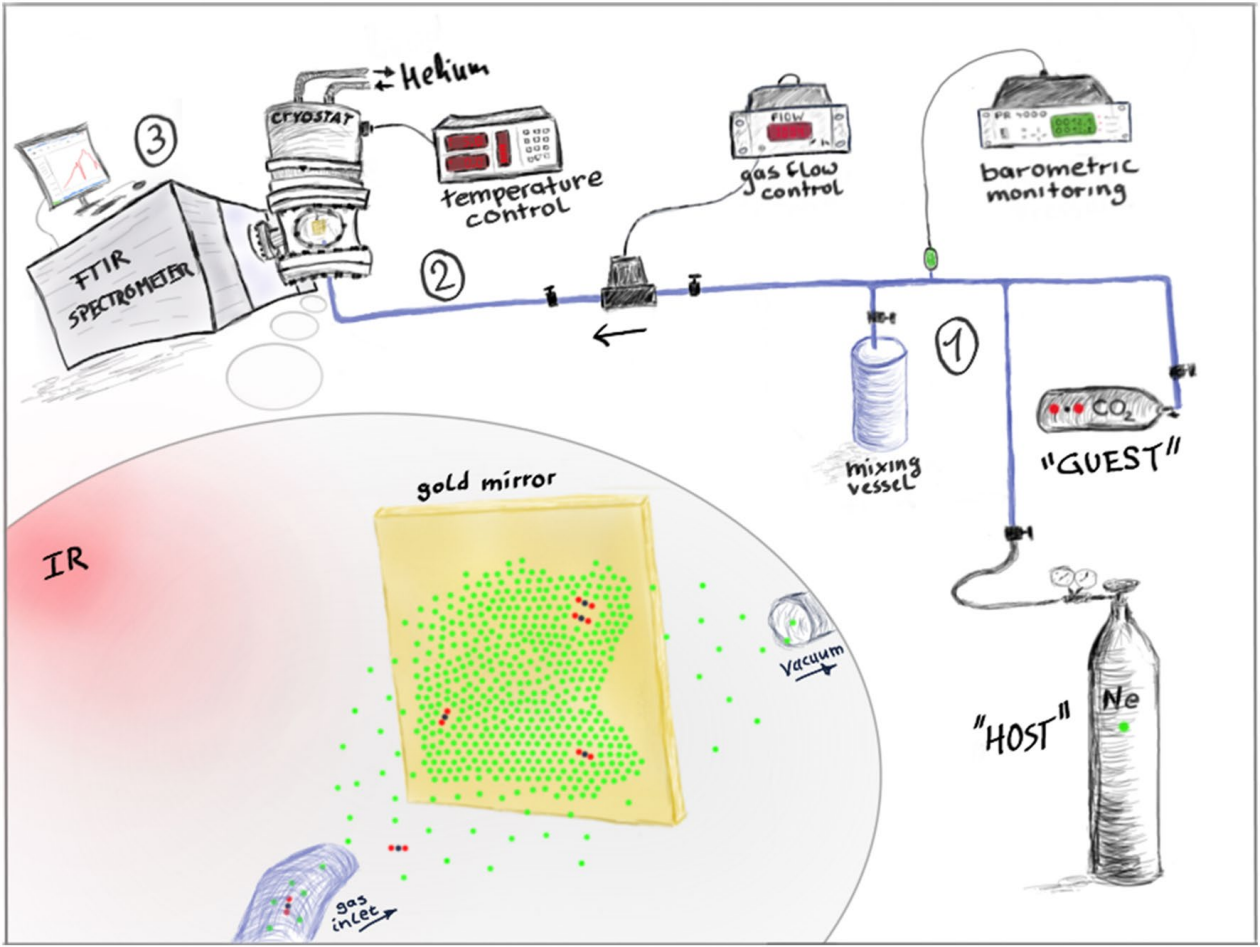
Sketch of the matrix-isolation technique for the use in IR spectroscopy, shown in three steps: (1) mixing of the host (e.g., Ne) with the guest (e.g., CO_2_) under barometric monitoring. (2) Deposition of the matrix by regulated gas flow into a liquid helium cooled cryostat. (3) Recording of the IR spectrum with a conventional FTIR spectrometer. Step (2) is sketched separately in the bottom left of the figure. The dimensions of the atoms are exaggerated

Although the fundamental idea is simple, matrix-isolation infrared (MI-IR) spectroscopy has a relatively elaborate experimental setup. Without too much detail, we will treat the experimental setup as consisting of three segments (Fig. 2): A mixing system, a cryostat, and a spectrometer. The mixing system (blue) comprises steel pipelines and a steel vessel (here 2 L). It is kept under a high vacuum (here at 10^–5^ mbar) and at room temperature (approx. 298 K). The cryostat operates at ultra-high vacuum (here at 10^–9^ mbar) and under cryogenic conditions (here at 5.8 K using a Gifford–McMahon cooler). A PID controller regulates the temperature in the cryostat. The FTIR spectrometer (here a Bruker Vertex 80v) operates under a high vacuum (10^–4^ mbar), and a vessel filled with liquid nitrogen cools the IR detector.

Figure 2 sketches a simplified MI-IR setup for the isolation of a stable, volatile analyte. For the sake of clarity, we omit the depiction of vacuum pumps, manometers, filters, and compressors. We focus on devices that are needed to comprehend the necessary steps and describe these devices in a simplified manner. Three steps may describe a basic MI-IR experiment:

1. A small amount of the analyte (here CO_2_) is expanded into the mixing vessel and stored therein. After the evacuation of the residual mixing system, the host gas (here Ne) is transferred into the same vessel. The mixing ratio of analyte and host is adjusted by barometric monitoring (e.g., 1 mbar of CO_2_ diluted in 1000 mbar of Ne). The gas mixture is stored within the mixing vessel to allow for diffusion while evacuating the residual mixing system.
2. The mixture slowly expands into the cryostat under controlled gas flow, e.g., at 0.8 mbar/min. Figure 2 sketches a close-up of the interior of the cryostat. A part of the instreaming gas mixture is pumped off, while another part deposits onto a gold mirror mounted within the cryostat and continuously cooled (here to 5.8 K). As the path length from the gas inlet to the gold mirror is short, the gas mixture does not change its composition significantly. The term “matrix” refers to the solid that forms on the gold plate. It cannot be in general foreseen, whether the matrix is crystalline or amorphous.
3. After matrix deposition, interrupting the gas flow and evacuating of the cryostat ensure that no residual gas mixture remains. IR radiation should only probe the deposited solid. As the host is IR inactive, only the guest molecules are visible in the IR spectrum of the matrix. These guest molecules are isolated, and their translational and rotational degrees of freedom are frozen. Hence, the observed bands are due to non-rotating vibrational transitions of single molecules.

## Results and discussion

### Computational assessment on the example of methanol

In the introduction, we have pointed out that the *accuracy of the PES* is at the center of each VSCF/VCI calculation. It is reasonable to assume that an accurate PES has a favorable effect on the overall accuracy of the VSCF/VCI calculation. However, it is a point of discussion to quantify the accuracy of the PES. The setup in the construction of the multimode PES influences its accuracy. It can be somewhat anticipated that the higher the level of electronic structure, the more accurate the PES. On the other hand, the accuracy of the PES can be assessed by comparison of the VSCF/VCI results with experimental data sets. In Table 1, we accumulate the results of such an assessment on the example of the ^12^CH_3_^16^OH isotopologue of methanol, where we investigate the prediction of (a) fundamental vibrational frequencies and (b) structural parameters including rotational constants, bond lengths and angles.

**Table 1:** Computational assessment of the accuracy of computed fundamental vibrational frequencies and structural parameters using different PES setups on the example of methanol (^12^CH_3_^16^OH)

Computational setups^a^	(a) Frequencies^b^				(b) Structural parameters^d^				
		MAD^c^/cm^−1^				MRD^e^/%			
		Ar	Ne	Gas		ED^f^	ED and MW^g^	MW and THz^h^	MMW and THz^i^
**Setup S1**	*ν*^Harm^	79.2	85.1	77.1	*r*_e_^BO^	1.24	1.16	0.61	0.58
1D: CCSD(T)/aug-cc-pVTZ	*ν*^VSCF^	26.4	29.2	25.7	*r*_g_^VSCF^	0.32	0.89	1.01	1.62
2D: MP4(SDQ)/aug-cc-pVTZ	*ν*^VCI(4)^	11.5	8.6	9.4	*r*_g_^VCI(4)^	0.47	0.97	1.10	1.42
3D and 4D: MP2/aug-cc-pVTZ	*ν*^VCI(5)^	7.6	5.7	6.9	*r*_g_^VCI(5)^	0.46	0.95	1.08	1.45
**Setup S2**	*ν*^Harm^	80.2	85.0	78.1	*r*_e_^BO^	1.27	1.31	0.72	0.78
1D: CCSD(T)-F12/cc-pVTZ-F12	*ν*^VSCF^	23.9	27.4	23.4	*r*_g_^VSCF^	0.25	0.67	0.72	0.97
2D: DCSD-F12/cc-pVTZ-F12	*ν*^VCI(4)^	12.0	9.6	10.0	*r*_g_^VCI(4)^	0.38	0.76	0.80	0.78
3D and 4D: AM1(repar.)/cc-pVTZ-F12	*ν*^VCI(5)^	10.7	8.4	7.9	*r*_g_^VCI(5)^	0.38	0.75	0.79	0.81
**Setup S3**	*ν*^Harm^	77.2	82.0	75.2	*r*_e_^BO^	1.27	1.30	0.71	0.77
1D and 2D: CCSD(T)-F12/cc-pVTZ-F12	*ν*^VSCF^	25.1	28.3	24.7	*r*_g_^VSCF^	0.25	0.66	0.70	0.91
3D and 4D: CCSD(T)-F12/cc-pVDZ-F12	*ν*^VCI(4)^	11.0	9.1	8.0	*r*_g_^VCI(4)^	0.40	0.77	0.78	0.71
	*ν*^VCI(5)^	6.7	2.9	3.8	*r*_g_^VCI(5)^	0.39	0.75	0.77	0.74
**Setup S4**	*ν*^Harm^	81.3	86.2	79.3	*r*_e_^BO^	1.37	1.51	0.92	1.11
1D and 2D: ae-CCSD(T)-F12/cc-pCVTZ-F12	*ν*^VSCF^	25.8	28.8	25.5	*r*_g_^VSCF^	0.24	0.67	0.56	0.64
3D and 4D: ae-CCSD(T)-F12/cc-pCVDZ-F12	*ν*^VCI(4)^	11.2	11.6	8.6	*r*_g_^VCI(4)^	0.47	0.74	0.69	0.49
	*ν*^VCI(5)^	6.5	5.6	4.0	*r*_g_^VCI(5)^	0.37	0.76	0.64	0.50
**Setup S5**	*ν*^Harm^	58.9	62.8	56.8	*r*_e_^BO^	1.14	1.04	0.54	0.42
1D–4D: B3LYP/aug-cc-pVTZ	*ν*^VSCF^	25.7	30.1	27.0	*r*_g_^VSCF^	0.26	0.82	0.95	1.21
	*ν*^VCI(4)^	18.2	15.7	18.2	*r*_g_^VCI(4)^	0.42	0.92	1.04	0.97
	*ν*^VCI(5)^	20.5	20.9	22.6	*r*_g_^VCI(5)^	0.41	0.90	1.03	1.04

Other computational approaches		Ar	Ne	Gas
Fast-VSCF/VCI (Scribano et al.^j^)		29.7	31.8	28.8
MM-RPH (Bowman et al.^k^)		7.9	7.5	6.6
cc-VSCF (Urena et al.^l^)		44.3	51.1	46.1
VV/VC (Sibert et al.^m^)		6.3	7.2	5.2
VPT2 (Miani et al.^n^)		7.7	8.9	10.6

^a^Setups differ in the electronic structure theory for one-mode (1D), two-mode (2D), three-mode (3D) and four-mode (4D) potentials

^b^Harmonic frequencies *ν*^Harm^ at the level of theory used for the 1D potential in the setup. VSCF frequencies *ν*^VSCF^ using up to 3D potentials. VCI Frequencies *ν*^VCI(4)^ (*ν*^VCI(5)^) with up to quadruple (quintuple) using up to 3D potentials (4D potentials)

^c^Mean absolute deviation (MAD) of computed frequencies w.r.t. our MI-IR data (Ar, Ne) and the revised gas-phase data accumulated by Perchard et al. [28]. The 11 fundamental frequencies in the range of 4000–500 cm^−1^ are considered, leaving out the torsion mode ν_12_

^d^Equilibrium parameters *r*_e_^BO^ from geometry optimization in the Born–Oppenheimer approximation at the level of theory used for the 1D potential in the setup. Vibrationally averaged parameters *r*_g_ from VSCF, VCI(4), and VCI(5)

^e^Mean relative deviation (MRD) of the structural and spectroscopic parameters w.r.t. gas-phase reference data from electron diffraction (ED), microwave (MW), submillimeter wave or terahertz (THz), and millimeter wave (MMW) experiments

^f^Averaged structural parameters (bond lengths, angles) derived from ED data by Benston et al. cf. details in Ref. [58]

^g^Zero-point averaged rotational constants, bond lengths and angles derived from ED and MW data by Iijiama, cf. details in Ref. [56]

^h^Rotational constants, bond lengths and angles derived from MW and THz data using isotope substitution by Gerry et al., cf. details in Ref. [59]

^i^Effective rotational constants derived from THz and MMW data by Herbst et al., cf. details in Ref. [57]

^j^Uses curvilinear coordinates and up to two-mode couplings in the PES at CCSD(T)/aug-cc-pVTZ level of theory, cf. details in Ref. [64]

^k^MM-RPH is based on VSCF/VCI. A full-dimensional semi-global PES at CCSD(T)/aug-cc-pVTZ level of theory is used, cf. details in Ref. [63]

^l^Uses up to two-mode couplings in the PES at MP2/TZV level of theory, cf. details in Ref. [62]

^m^Perturbative/variational approach relying on anharmonic quartic force-field at CCSD(T)/cc-pVTZ level of theory, cf. details in Ref. [61]

^n^Perturbative approach relying on anharmonic quartic force field at MP2/cc-pVTZ lever of theory, cf. details in Ref. [60]

The computational assessment shown in Table 1 comprises an extensive statistical analysis of the accuracy of five different PES setups in our calculations. Additionally, we compare our results to a variety of previous calculations regarding the fundamental vibrational frequencies [60–64]. Details of the PES setups (S1–S5) are listed together with the results. Setup S1 uses conventional coupled-cluster theory CCSD(T) and perturbation theory MP4(SDQ), respectively, MP2. Setup S2 uses explicitly correlated coupled-cluster CCSD(T)-F12 [52] and distinguishable cluster theory DSCD-F12 [53, 54]. Furthermore, it employs a semiempirical reparameterization scheme [51]. Setup S3 and S4 use the explicitly correlated coupled-cluster theory CCSD(T)-F12 [52]. In setup S1 and S2, the level of electronic structure theory is hierarchically decreased from 1 to 4D potentials, using the same basis set in for all potentials. In setup S3 and S4, the level of electronic structure theory is maintained for all potentials, while basis sets change from triple-ζ (1D and 2D potentials) to double-ζ (3D and 4D potentials). Setup S5 uses the B3LYP density functional as implemented in Molpro (exchange: 0.2 Hartree–Fock + 0.72 Becke88 [67] + 0.08 Slater-Dirac [68, 69], correlation: 0.81 Lee–Yang–Parr [70] + 0.19 Vosko–Wilson–Nusair functional V [71]).

#### Fundamental vibrational frequencies

For each PES setup, the computed harmonic frequencies (*ν*^Harm^), as well as VSCF and VCI frequencies (*ν*^VSCF^ and *ν*^VCI^), are compared to our Ne and Ar MI-IR data and the literature gas-phase IR data. For the latter, we rely on the gas-phase IR reference dataset accumulated by Perchard et al. [28] for the ^12^CH_3_OH isotopologue. In the left part of Table 1, the mean absolute deviation (MAD) measures the discrepancy between calculated and experimental in cm^−1^. The lower the MAD, the better the overall agreement with the experiment. We denote VCI calculations using up to 3D potentials and quadruple excitations in the CI space as VCI(4), and when using up to 4D potentials and quintuple excitations as VCI(5).

For the harmonic frequencies (*ν*^Harm^), setup S5 shows the best results (MAD ≈ 60 cm^−1^), while setups S1–S4 are slightly worse (MAD ≈ 75–85 cm^−1^). It is common to all setups that the anharmonic frequencies (*ν*^VSCF^,* ν*^VCI(4)^, and *ν*^VCI(5)^) with a MAD below 30 cm^−1^ are superior to *ν*^Harm^. However, this anharmonic correction is somewhat different for the various setups. For setup S5, *ν*^VSCF^ yields significant improvement (MAD ≈ 25–30 cm^−1^), while *ν*^VCI(4)^ and *ν*^VCI(5)^ bring only a relatively small correction (MAD ≈ 20 cm^−1^). In other words, VCI does not impose a vivid correction upon VSCF when using setup S5. This result is in remarkable contrast to the other setups (S1–S4). For the latter setups, *ν*^VSCF^ is similar to setup S5 (MAD = 25–30 cm^−1^). However, *ν*^VCI(4)^ brings significant improvement (MAD ≈ 10 cm^−1^) of a factor between 2 and 3 compared *ν*^VSCF^. To give a particular example: Using setup S3 the MAD w.r.t. gas-phase data reduces from 24.7 cm^−1^ (*ν*^VSCF^) to 8.0 cm^−1^ (*ν*^VCI(4)^), corresponding to an improvement factor of 3. Similar statements hold for setups S1, S2, and S4.

Considering setups S1 and S2, the MADs decrease only slightly when 4D potentials instead of 3D potentials are incorporated, i.e., *ν*^VCI(5)^ is only slightly better than *ν*^VCI(4)^. In contrast to that, based on the setups S3 and S4, the *ν*^VCI(5)^ frequencies are in excellent agreement with the experiment (MAD ≈ 5 cm^−1^). Note that this good agreement holds for all the different experimental IR references (gas-phase, Ne, and Ar). In other words, *ν*^VCI(5)^ relying on setups S3 and S4 shows an improvement factor between 5 and 6 compared to *ν*^VSCF^, and an improvement factor of 20 compared to *ν*^Harm^. Consequently, setups S3 and S4 resemble the best overall agreement with experiment when it comes to fundamental vibrational frequencies.

In the past, a variety of computational approaches have been studied regarding the fundamental vibrations of the methanol molecule. We consider three studies using variational approaches (fast-VSCF/VCI [64], MM-RPH [63], cc-VSCF [62]) that are in parts similar to the approach in this work. Furthermore, we mention one study using a perturbative approach (VPT2 [60]) and another using a mixture of perturbative and variational approaches (VV/VC [61]). Within these approaches, the VV/VC results show the best agreement with the experiment (MAD ≈ 6 cm^−1^). The results from our VSCF/VCI calculations using setup S3 or S4 are slightly better (MAD ≈ 5 cm ^−1^) than the referenced VV/VC results. Also, the referenced VPT2 and MM-RPH results are in good (MAD ≈ 9 cm^−1^) and very good (MAD = 7 cm^−1^) agreement with the experiment. The referenced fast-VSCF/VCI and cc-VSCF results, however, show rather poor agreement (MAD ≈ 30 cm^−1^ and MAD ≈ 45 cm^−1^) with the experiment.

#### Structural parameters

The right part of Table 1 compares computed equilibrium structural parameters (*r*_e_) and vibrationally averaged structural parameters (*r*_g_) with various literature datasets from gas-phase experiments [56–59]. We use four datasets as reference: Rotational constants, bond lengths, and angles derived from a combination of microwave and terahertz (MW and THz) data using isotope substitution by Gerry et al. [59]. Zero-point averaged rotational constants, bond lengths, and angles derived from a combination of electron diffraction and microwave (ED and MW) data by Iijiama [56]. Effective rotational constants derived from a combination of terahertz and millimeter-wave (THz and MMW) data by Herbst et al. [57]. Finally, averaged bond lengths and angles derived from electron diffraction (ED) data by Benston et al. [58].

The computation includes Born–Oppenheimer equilibrium structural parameters (*r*_e_^BO^) as well as vibrationally averaged structure parameters based on VSCF (*r*_g_^VSCF^) and VCI (*r*_g_^VCI(4)^ and *r*_g_^VCI(5)^). The structural parameters (rotational constants, bond lengths, and angles) are on different scales. Thus, the assessment relies upon mean relative deviation (MRD in %) of the computed parameters compared to the experimental, rather than absolute deviations. In general, the observed MRDs are in the range of 0.3–1.7%. Although these values may not seem significant, they are. The following should demonstrate that for a seemingly small MRD, the absolute deviation is rather significant. The CO bond has an experimentally measured bond length of 1.424 Å. An MRD of 1.7% means that calculation predicts the CO bond length in a range of 1.448–1.400 Å. An MRD of 0.3% means that this bond length is predicted between 1.428 and 1.420 Å. The latter is a significantly better prediction. Table S1 in the electronic supplementary information provides all absolute values for the various structural parameters from theory and computation.

When compared to pure ED data or a mixture of ED and MW data, the vibrationally averaged structural parameters (*r*_g_^VSCF^ and *r*_g_^VCI^) are generally superior to the equilibrium structural parameters (*r*_e_^BO^). Using any of the five PES setups (S1–S5), the MRD is lower for *r*_g_ than for *r*_e_, with setup S4 providing the best agreement with experiment. The equilibrium parameters *r*_e_^BO^ as computed based on setup S4, show an MRD of 1.51% w.r.t. ED data, respectively, 1.37% w.r.t. ED and MW data. Using the same setup S4, the vibrationally averages parameters *r*_g_^VCI(5)^ show an MRD of 0.37% w.r.t. ED data and an MRD 0.76% w.r.t. ED and MW data. In other words, the agreement with the experiment is better for *r*_g_^VCI(5)^ than for *r*_e_. Compared to the ED dataset, it is 3.7 times better, compared to the ED and MW dataset it is 2.0 times better.

Note that contradictory results are observed with the other experimental datasets as reference. That means *r*_g_^VSCF^ and *r*_g_^VCI^ are not generally superior to *r*_e_^BO^, when comparing the calculated parameters to a mixture of MW and THz data or a mixture of MMW and THz data. Relying on setups S1 and S5, *r*_e_ shows a significantly lower MRD than *r*_g,_ and using the setups S2 and S3, *r*_e_ shows a very similar MRD as *r*_g_. Only for setup S4 the MRD of *r*_g_ is lower, thus better, than of *r*_e_. When the structural parameters are computed based on setup S4, the resulting *r*_g_^VCI(5)^ agrees 1.4 times better (MW and THz data), respectively, 2.2 times better (MMW and THz data) than *r*_e_^BO^ to the experiment.

In general, we observe with very few exceptions that *r*_g_^VCI(5)^ shows slightly better MRD than *r*_g_^VCI(4)^. Surprisingly, *r*_g_^VSCF^ often shows an even better MRD than *r*_g_^VCI(5)^. However, the differences are rather small. Finally, *r*_g_^VCI(5)^ using PES setup S4 shows the most consistent and best overall agreement with various gas-phase ED, MW, MMW, and THz experimental data.

#### Findings from the computational assessment

When it comes to fundamental vibrational frequencies (cf. MAD in Table 1), the discrepancy between theory and experiment systematically diminishes when going from the harmonic approximation (*ν*^Harm^) to vibrational self-consistent field (*ν*^VSCF^) and vibrational configuration interaction (*ν*^VCI(5)^). These findings confirm that anharmonicity and mode-coupling in the multimode PES and the subsequent VSCF/VCI calculation are a rigorous step beyond the harmonic approximation. Furthermore, using coupled-cluster theory in the computation of the electronic structure provides the most accurate PES. Given an accurate PES with up to 4D potentials, the VSCF/VCI calculations presented here are as functional as previous calculations (MM-RPH [63], VPT2 [60], and VV/VC [61]), or even better (fast-VSCF/VCI [64], cc-VSCF [62]). This comparison must be, however, appreciated with care. Both the fast-VSCF/VCI computations and the cc-VSCF using up to 2D potentials, which may be the reason why these calculations show relatively poor results. It could be expected that the authors would have reached somewhat better results by further expanding their PES.

We obtain the best results using a PES of up to 4D potentials and the setup S3 or S4, relying on the explicitly correlated coupled-cluster approach CCSD(T)-F12 with triple-ζ basis sets in the 1D and 2D potentials and double-ζ in 3D and 4D potentials. Setup S3 and S4 yield slightly better results than setup S1, which relies on the conventional coupled-cluster approach CCSD(T) in the 1D potentials, perturbation theory MP4 for the 2D potentials (resp. MP2 for the 3D and 4D potentials), all with triple-ζ basis sets. These results indicate that (1) the use of coupled-cluster theory is superior to perturbation theory, and (2) the use of explicit correlation is preferable. The latter was to be expected because the explicitly correlated F12 approaches used in setup S3 or S4 provide a better basis-set convergence that their conventional counterpart. Furthermore, an all-electron correlation treatment is preferable. We have seen that setup S4, which considers the correlation between valence-shell and core electrons, yields slightly better results than setup S3, where electron correlation is considered only for the valence-shell.

The results obtained by setup S2 are reasonably good. In this particular setup, however, incorporation of 4D potentials does not bring similar improvements as observed for the incorporation of 4D potentials in setups S1, S3, and S4. When using setup S2, we observe that VCI(5) based on up to 4D potentials yields only slightly better results than VCI(4) based on up to 3D potentials. Although the accuracy is not excellent for this setup, it has some benefits when it comes to computational costs. Setup S2 uses CCSD(T)-F12 in the 1D potentials and DCSD-F12 in the 2D potentials, while the 3D and 4D potentials are computed by a reparametrized semiempirical AM1 approach [51]. As an AM1 calculation takes only seconds, the computation time of 3D and 4D potentials is dramatically reduced compared to setup S1, S3, and S4. Besides, the DCSD-F12 approach is very efficient and seems to produce accurate 2D potentials without computation of perturbative triples (T).

Contrary to the evaluation of the fundamental vibrational frequencies, our results for structural parameters (cf. MRD in Table 1) do not necessarily imply a uniform trend. In this respect, we cannot conclude that the discrepancy between theory and experiment systematically diminishes when going from computed equilibrium parameters *r*_e_ to vibrationally-averaged parameters *r*_g_. On the contrary, the discrepancy highly depends on the experimental reference. In general, our results show that vibrationally-averaged parameters *r*_g_ resemble electron diffraction (ED) data better than spectroscopic data (MW, MMW, THz). On the other hand, the equilibrium parameters *r*_e_ resemble spectroscopic data better. Nevertheless, setup S4 consistently yields the best overall agreement with all experimental data. Hence, we may conclude that the incorporation of all-electron correlation and explicit correlation is preferable when aiming at proper structural parameters.

Setup S5, using the B3LYP density functional, is in vivid contrast to all the other setups. For *ν*^Harm^, setup S5 is in better agreement with experiment than all CCSD(T) approaches used in this study, yet, a worse agreement with experiment regarding *ν*^VSCF^ and *ν*^VCI(5)^. That implies that density functional theory is insufficient for the construction of a useful multimode PES for the accurate calculation of anharmonic spectra by VSCF/VCI. Additionally, it is remarkable that setup S5 is always better than all other setups when it comes to equilibrium parameters *r*_e_, no matter which experimental data function as a reference. These observations may be reasoned as follows. The parametrization of the B3LYP functional against experimental datasets includes reference systems that are in their geometrical equilibrium structure. Thus, the B3LYP functional performs well in describing electron correlation for the equilibrium structure, yet, it may fail in describing electron correlation far from the equilibrium. In the construction of the multimode PES, however, also non-equilibrium conformations must be described. All our results show that an accurate description of electron correlation outside the equilibrium geometry is essential in the construction of the PES. When constructing a PES by computing the electronic structure with an approach initially parametrized toward the equilibrium structure, this PES will yield poor results in subsequent VSCF/VCI calculations. The methanol molecule itself is part of the experimental dataset for the least-squares fit in Becke’s publication from 1993 [72], which is the origin of the typical B3LYP implementation later formulated by Stephens et al. [73]. Thus, it may not be surprising that the equilibrium parameters *r*_e_ calculated for methanol with the B3LYP functional shows such good agreement with almost all experimental datasets.

### Case studies on the synergy of MI-IR and VSCF/VCI

We performed multiple MI-IR experiments on stable, small molecules, such as water [29], carbon dioxide, and methane [30], fluoroethane [74], methanol, and others. In all those studies, we use argon and neon as host systems and utilize various dilutions and temperature changes to learn about the influence of matrix effects. We observe in the majority of cases that the vibrations of a molecule trapped in highly diluted neon matrices at 6 K are very similar to the vibrations of this molecule in gas phase. On the contrary, trapping in argon matrices sometimes introduces quite substantial matrix effects. Moreover, the matrix effects in different host–guest systems tend to be very unsystematic.

**Fig. 3:**
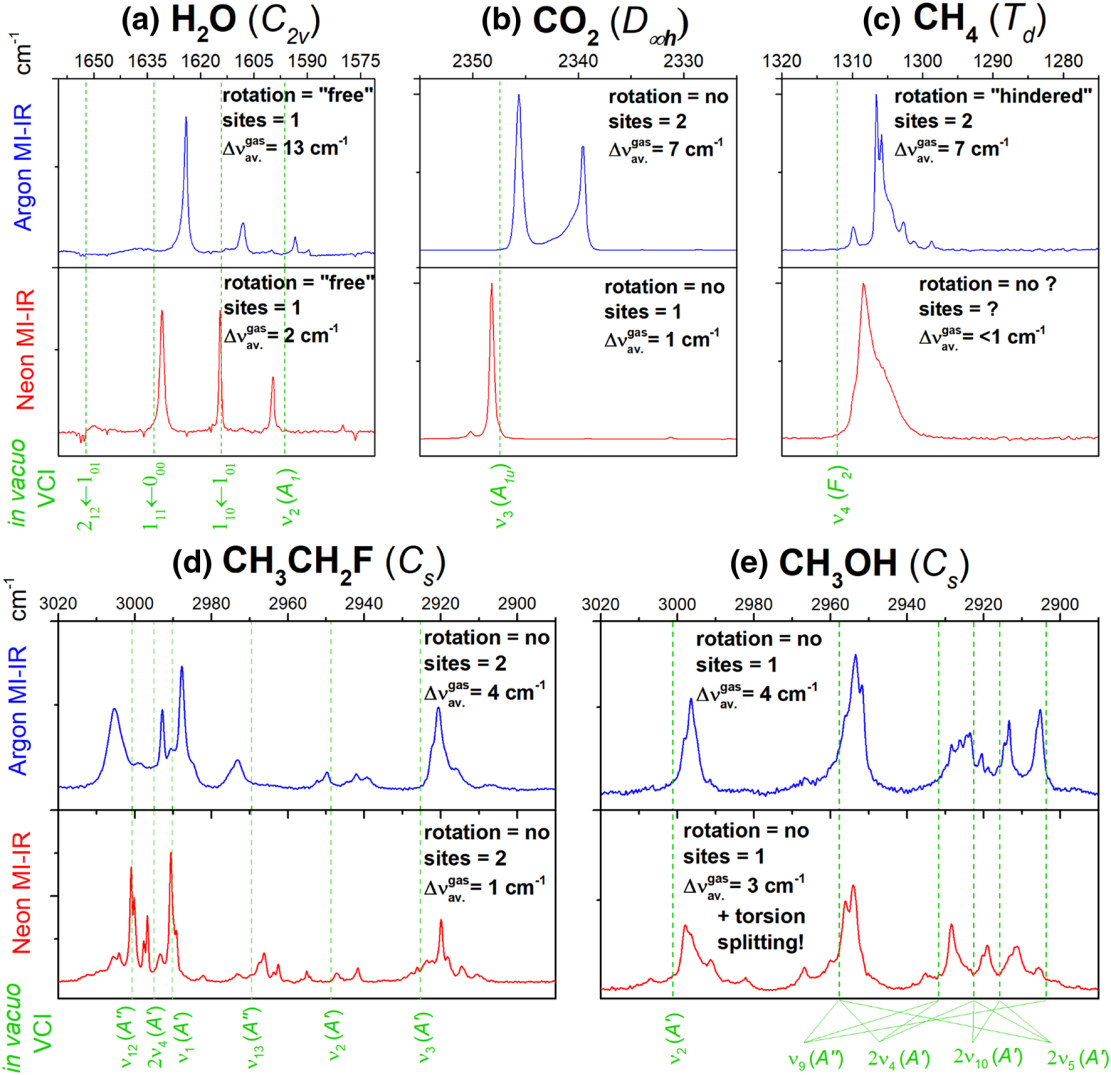
Selected regions of the MI-IR spectra of **a** water (H_2_O), **b** carbon dioxide (CO_2_), **c** methane (CH_4_), **d** fluoroethane (CH_3_CH_2_F), **e** methanol (CH_3_OH), as trapped with high dilution (< 500 ppm) in argon (blue) and neon (red) matrices, together with *in vacuo* VCI calculated spectra (dashed green) based on multimode PESs with CCSD(T)-F12/VTZ-F12 level of electronic structure theory. Each panel contains information about matrix effects, i.e., rotation, trapping sites, and averaged matrix shifts from the gas phase ${{\Delta \nu }}_{\mathrm{av}.}^{\mathrm{gas}}$ (mean absolute deviation of the fundamental vibrational transitions between the observed MI-IR data and gas-phase data from literature). The assignments (green labels) are molecule specific and rely on the VCI calculated transitions. The scale of the experimental IR intensities is not comparable among the different experiments

For an evaluation of the synergy of MI-IR spectroscopy and *in vacuo* VCI computation, the matrix effects must be well-understood. The following sections present central aspects that have emerged so far from our studies. We deal with the matrix effects observed for some of the before-mentioned molecules and shed light on the agreement between the MI-IR spectrum and the VCI calculated spectrum. Figure 3 presents these aspects in a selection of MI-IR spectra, together with the corresponding VCI calculated energy levels. We intentionally show only parts of each spectrum and keep our discussion in a greater context, rather than analyzing the spectra independently in-detail. More experimental details (isotopic labeling, dilutions, and temperature changes) and VCI results are available in our original publications mentioned above.

#### Water: revised spectral assignments in a well-studied molecule

The investigation on the monomers of water (H_2_^16^O, HD^16^O, and D_2_^16^O) [29] shows a matrix shift (${{\Delta \nu }}_{\mathrm{av}.}^{\mathrm{gas}}$) for argon of about 13 cm^−1^ and for neon of roughly 2 cm^−1^. Additionally, we observe a very systematic band splitting pattern in the spectrum (cf. Fig. 3a). These bands cannot be matrix splittings because the number of bands would imply an unreasonable amount of trapping sites. In contrast, multiple trapping sites would cause band shapes like the doublet observed for carbon dioxide in argon (cf. Fig. 3b).

Early MI-IR studies of water have already assumed that it rotates in the matrix [3–6]. Because of the low temperature, only a few rotational–vibrational transitions are observable. Figure 3a exemplifies the fundamental bending vibration *ν*_2_(*A*_1_), for which we see in the MI-IR spectra at least three rotational–vibrational transitions, i.e., 2_12 _← 1_01_, 1_11 _← 0_00_, and 1_10 _← 1_01_. Those denote the transition from the initial state *J*″_Ka″,Kc″_ to the final state *J*′_Ka′,Kc′_, where *J* is the rotational quantum number and Ka, Kc the projection quantum numbers of the asymmetric top. The rotational–vibrational line spacings observed in the matrix are similar to their counterpart in the gas phase. Hence, rotation in the matrix is similar to the free rotation in the gas phase.

The tendency to form dimers is rather high for water, and those dimers are observed in the matrix. Although even in Ne matrices, most dimer bands of water are well-known [5], we were able to assign new HDO dimers and mixed dimers [29]. We observed oligomerization also in carbon dioxide, methane, methanol, and fluoroethane. Although oligomerization occurs in all host–guest systems, the tendency to form oligomers strongly depends on the guest molecule. Because water and methanol can form hydrogen bonds, they tend to form oligomers at rather high dilutions, whereas methane, carbon dioxide, and fluoroethane exhibit relatively weak oligomer bands at high dilutions. Howsoever, oligomerization is a minor issue when it comes to identifying matrix effects. It is always possible to minimize the occurrence of oligomers by increasing dilution.

For the VCI calculated vibrational transitions, perfect agreement with gas-phase data was observed, with a deviation of roughly 1 cm^−1^. The discrepancies between VCI calculated spectra and MI-IR spectra (both Ar and Ne) are on the same scale as the discrepancies between the MI-IR and gas-phase spectra. Thus, residual discrepancies are caused by neglecting matrix effects in the calculations. The adiabatic rotation approximation (ARA) [75] approximately calculates the rotational–vibrational transitions. In our MI-IR experiments, we mainly observed *J* = 1 and *J* = 2, for which the VCI + ARA computations yielded consistent results with deviations of roughly 13 cm^−1^ in Ar and 3 cm^−1^ in Ne. This discrepancy is supposedly due to matrix effects. However, we expect improvement from computing the rotational–vibrational states in a more sophisticated way, e.g., by rotational–vibrational configuration interaction (RVCI) [76]. In the following, we will see that methane also rotates in the matrix. However, this rotation is not comparable to the free rotation of methane in the gas phase. Hence, VCI + ARA calculations as we performed for the water molecule would not be reasonable for the methane molecule. Moreover, also RVCI calculations of gas-phase methane will not be comparable to the MI-IR spectrum of methane.

#### Carbon dioxide and methane: matrix effects are not systematic

Similar to water, the VCI calculation of both carbon dioxide (CO_2_) and methane (CH_4_ and CD_4_) yields excellent agreement with the experiment. The discrepancy between VCI calculation and gas-phase data is roughly 1 cm^−1^ for carbon dioxide and roughly 4 cm^−1^ for methane. These discrepancies are again similar to the matrix shifts. For both carbon dioxide and methane, ${{\Delta \nu }}_{\mathrm{av}.}^{\mathrm{gas}}$ is 7 cm^−1^ in argon and roughly 1 cm^−1^ in neon. Consequently, the VCI calculations are very well suited to assign the vibrational transitions in the MI-IR experiments. Notice that the matrix shift in argon is 2 times larger for water than for carbon dioxide and methane.

The occurrence of matrix splittings due to multiple trapping sites is not immediately detectable in Ar matrices of water and methane. However, for carbon dioxide, at least two trapping sites can be assigned to the very pronounced double peak (cf. Fig. 3b). For all three molecules, there is generally no need to impose different trapping sites in Ne matrices, because we do not observe any systematic splittings that need this interpretation. In contrast to that, in the case of fluoroethane, at least two trapping sites must be imposed to interpret the observed spectrum.

Carbon dioxide does not show evidence of rotation in the MI-IR spectrum, neither in Ne nor in Ar matrices. The spectra of carbon dioxide (cf. Fig. 3b) exhibit no systematic band splitting pattern that could arise from rotational–vibrational transitions. For methane, however, there is a prominent splitting pattern in the AR MI-IR spectrum (cf. Fig. 3c), and also a relatively broad band in the Ne MI-IR spectrum. As stated above, water rotates almost freely in matrices. The corresponding rotational–vibrational transitions show large spacings (cf. Fig. 3a) similar to gas-phase water. Methane rotation must be hindered in matrices because the observed pattern (cf. Fig. 3c) has way smaller spacings than known from the rotational–vibrational transitions of gas-phase methane.

This interpretation represents a minimum consent from our work and a variety of studies on carbon dioxide [12, 14, 15, 77] and methane [17, 20, 22, 78] that date back to the 1960s. However, especially the history of spectral assignments of CO_2_, CH_4_, and CD_4_ shows that the interpretation of their MI-IR spectra is very controversial. We have recently shown that the matrix effects for these two molecules in different host materials are far from being systematic [30] and that methane might also rotate in Ne matrices.

It is striking how different the matrix effects for small molecules are, considering their geometrical simplicity. These observations underscore the assumption that there is an intricate link between matrix effects and the interplay of host and guest at cryogenic conditions. Argon as a host imposes more pronounced matrix effects then neon. In a first naïve concept, one may argue that the electron shell of neon is much smaller than of argon, making its interaction with the trapped analyte less pronounced. While this naïve interpretation may be successful in conceptualizing the matrix shifts, other matrix effects (trapping sites, rotation) are not as simple to interpret. To overcome this, one may introduce concepts considering the host–guest structure of argon and neon together with the analyte. However, it is not straightforward to predict such structures, and they may not be transferable from one molecule to another. For now, it looks like matrix effects in MI-IR spectroscopy are too diverse to be easily grasped in a single theoretical concept applicable to different host–guest systems.

#### Fluoroethane: elucidation of the CH stretch region

Considering fluoroethane (ethyl fluoride, HFC-161), we have exemplified an application of the interplay of MI-IR and VSCF/VCI [74]. HFC-161 is a hydrofluorocarbon increasingly used in refrigerant mixtures. Although such mixtures have lower ozone-depletion potentials compared to previously used chlorofluorocarbons, they absorb significantly in the atmospheric IR window, making them potential greenhouse gases. Thus, IR spectroscopic characterization of HFCs is of high importance to understand their global warming potential. A vital issue in the assignment of the gas-phase IR spectrum of fluoroethane is the overlap of rotational–vibrational fine structure. In other words, gas-phase data do not provide sufficient information to assign all fundamental vibrations and to evaluate the theoretical predictions.

Thus, we recorded Ar and Ne MI-IR spectra of CH_3_CH_2_F and CD_3_CD_2_F and accomplished an assignment of all fundamental vibrations with the help of extensive VSCF/VCI calculations. The assignment has been especially challenging in the CH and CD stretch region, where literature data did not provide any sensible distinction of the ν_1_, ν_12_, and ν_13_ fundamentals. Based on our combined experimental and computational approach, we have been able to solve this assignment (cf. Fig. 3d). The observed Ar matrix shift is about 4 cm^−1^, while the Ne matrix shift is roughly 1 cm^−1^. Both Ar and Ne MI-IR spectra show systematic matrix splitting patterns suggesting at least two trapping sites. We found no evidence for the rotation of the fluoroethane monomer in the matrix. Finally, some bands can be explained by the aggregation to dimers or higher oligomers. However, fluoroethane is far less prone to aggregation than water or methanol.

Similar to the results of the above computational assessment, we also observe for the fluoroethane molecule that the VCI results tend to be in better agreement with the experiment when computing CCSD(T)-F12 single points with all-electron correlation than with valence-electron correlation only. The evidence for this is that the discrepancy of the CH stretch fundamentals as computed by VCI in comparison with either MI-IR or gas-phase IR minimizes when using all-electron correlation. Also, the predicted structural parameters agree better with previous gas-phase electron diffraction and microwave data. In short, in the case of fluoroethane, the mean absolute derivation between VCI and MI-IR has shown to be about 4 cm^−1^ for Ar matrices and about 2 cm^−1^ for Ne matrices.

#### Methanol: conventional spectroscopic notations are limited

The computational assessment (cf. Table 1) on the example of methanol (^12^CH_3_^16^OH) shows an excellent agreement between VCI calculation and experiment, both gas-phase and MI-IR data. Relying on an accurate PES, the deviations are below 3 cm^−1^. These deviations are similar to the matrix shifts. The observed Ar matrix shift is about 4 cm^−1^, while the Ne matrix shift is roughly 3 cm^−1^. In this respect, we can draw the same conclusion for methanol as for the previously discussed cases. The matrix shifts are systematic, while other matrix effects are not. In the MI-IR spectra of methanol, however, we encounter another issue. Where the harmonic approximation predicts only three fundamental vibrations, a multitude of bands occurs in the experiment (Fig. 3e). Even after eliminating possible origins arising from matrix effects, the number of unassigned bands remains still significant.

The assignment of overtones and combination bands of the CH deformation usually solves this issue. Such an assignment relies on the concept of dissecting molecular vibration into normal-mode coordinates. Most conventional notations in spectroscopy have their roots in this dissection. For example, the definition of the label “CH stretch” rests on assuming the existence of an intramolecular motion described by a rectilinear coordinate in the plane of the C-H bond. The concept of VCI calculations based on a multimode PES, however, couples the normal-mode coordinates and reverses the before-mentioned dissection. Our computational assessment has nicely demonstrated that this coupling of normal-mode coordinates is necessary for a correct quantitative agreement of computed vibrational frequencies with the experimentally observed ones. In other words, the dissection into normal-mode coordinates is not successful from a quantitative point of view. It remains to debate whether the dissection into normal-modes is also problematic from a qualitative point of view.

In Fig. 3e, we depict the contributions of different normal-modes to a VCI calculated state, as obtained from a preliminary analysis of the VCI calculations. As one may observe in this figure, the assignment is not unique, and an intricate web of lines depicts various possibilities. The fundamental CH stretch vibration *ν*_9_(*A*″) has contributions to at least three vibrational states. Similar statements hold for the overtones 2*ν*_4_(*A*′), 2*ν*_10_(*A*′), and 2*ν*_4_(*A*′). Furthermore, also combinations of *ν*_4_(*A*′), *ν*_10_(*A*′), and *ν*_4_(*A*′) (intentionally omitted in Fig. 3e) could label the observed bands in this spectral region. One may choose to renounce to a final notation of these problematic bands and be satisfied with the quantitative agreement between theory and experiment. However, it is vital to retain a notation for the assignment of experimental bands to communicate the observations made. Such notation is most comfortable in the picture of normal-mode coordinates, even though for a quantitative agreement with the experiment, the calculation must go beyond this picture. The best notation used to describe such bands, thus, remains an unsolved issue.

## Closing remarks

This minireview summarizes synergetic effects from the combination of MI-IR spectroscopy and VCI computations based on multimode PESs. We provide a computational assessment to highlight some basic computational settings for successfully facilitating the assignment of experimental IR spectra with the help of VCI computations. With a variety of case studies, we demonstrate the flexibility of the theoretical approach, which models a molecule *in vacuo* to compute its vibrations by VSCF/VCI on multimode PESs. Besides flexibility, also accuracy is ensured in this approach, shown by the excellent agreement compared to gas-phase data but also neon matrix-isolation data. The remaining discrepancies between the MI-IR experiment and the VCI computation are primarily due to matrix effects not grasped in the theoretical model. A theoretical conceptualization of matrix effects would be beneficial when the MI-IR experiment shall provide a physical interpretation of the host–guest structure. However, we have shown that matrix effects do not impede spectroscopic characterization. We expect that a combined theoretical and experimental procedure, as suggested here, will increase in popularity in the future. As the performance and accuracy of the VSCF/VCI approach are steadily improving, its application to a variety of molecules is foreseeable.

## Electronic supplementary material

Below is the link to the electronic supplementary material.

Supplementary file1 (DOCX 35 kb)

## Data Availability

The authors confirm that the data supporting the findings of this study are available within the article and its supplementary materials.
