# Supplementary material for: On the synergy of matrix-isolation infrared spectroscopy and vibrational configuration interaction computations
Source: Theor Chem Acc. 2020 Nov 9;139(12):174. doi: 10.1007/s00214-020-02682-0 (PMC7652801; doi:10.1007/s00214-020-02682-0)
Supplement: Supplementary file 1 — Supplementary file1 (DOCX 35 kb) [file 214_2020_2682_MOESM1_ESM.docx]

**Electronic Supplementary Information**

**On the Synergy of Matrix-Isolation Infrared Spectroscopy
and Vibrational Configuration Interaction Computations**

Dennis F. Dinu^1,2,3^, Maren Podewitz^1^, Hinrich Grothe^2^, Thomas Loerting^3^, Klaus R. Liedl^1^

^1^ Institute of General, Inorganic and Theoretical Chemistry, University of Innsbruck, Austria

^2^ Institute of Material Chemistry, TU Vienna, Austria

^3^ Institute of Physical Chemistry, University of Innsbruck, Austria

e-mail: klaus.liedl@uibk.ac.at

| **Table S1: Computed and experimental structural parameters used for the computational assessment.** | | | | | | | | | | | | |
| --- | --- | --- | --- | --- | --- | --- | --- | --- | --- | --- | --- | --- |
| **Setup** | **Parameter** | **A / GHz** | **B / GHz** | **C / GHz** | **r_CO_ / Å** | **r_OH_ / Å** | **α_COH_ / °** | **r_CH_ / Å** | **α_OCH_ / °** | **r_CH,oop_ / Å** | **α_OCH,oop_ / °** | **α_HCH_ / °** |
| **S1** | *r_e_^BO^* | 128.88530 | 24.73180 | 23.89726 | 1.424 | 0.960 | 109.7 | 1.089 | 109.7 | 1.089 | 109.7 |  |
|  | *r_g_^VSCF^(3D)* | 124.39204 | 24.34479 | 23.54341 | 1.435 | 0.960 | 108.4 | 1.100 | 106.8 | 1.106 | 111.9 |  |
|  | *r_g_^VCI(4)^* | 125.06807 | 24.34156 | 23.55778 | 1.435 | 0.951 | 108.8 | 1.100 | 106.9 | 1.106 | 111.8 |  |
|  | *r_g_^VSCF^* | 124.39603 | 24.34267 | 23.54246 | 1.435 | 0.959 | 108.4 | 1.100 | 106.8 | 1.106 | 111.9 |  |
|  | *r_g_^VCI(5)^* | 125.02211 | 24.33724 | 23.55216 | 1.435 | 0.951 | 108.6 | 1.100 | 106.8 | 1.106 | 111.9 |  |
| **S2** | *r_e_^BO^* | 128.80374 | 24.81405 | 23.97927 | 1.420 | 0.957 | 109.8 | 1.091 | 109.8 | 1.091 | 109.8 |  |
|  | *r_g_^VSCF^(3D)* | 125.26634 | 24.50078 | 23.69142 | 1.429 | 0.957 | 108.7 | 1.098 | 106.9 | 1.105 | 111.9 |  |
|  | *r_g_^VCI(4)^* | 125.88898 | 24.50015 | 23.70765 | 1.429 | 0.949 | 109.0 | 1.098 | 106.9 | 1.104 | 111.9 |  |
|  | *r_g_^VSCF^* | 125.27790 | 24.49560 | 23.68722 | 1.430 | 0.957 | 108.6 | 1.098 | 106.9 | 1.105 | 111.9 |  |
|  | *r_g_^VCI(5)^* | 125.86975 | 24.49235 | 23.69851 | 1.430 | 0.949 | 108.8 | 1.098 | 106.9 | 1.104 | 112.0 |  |
| **S3** | *r_e_^BO^* | 128.79373 | 24.81156 | 23.97696 | 1.420 | 0.957 | 109.8 | 1.091 | 109.8 | 1.091 | 109.8 |  |
|  | *r_g_^VSCF^(3D)* | 125.29922 | 24.52083 | 23.70823 | 1.429 | 0.957 | 108.7 | 1.099 | 106.9 | 1.104 | 111.9 |  |
|  | *r_g_^VCI(4)^* | 125.99155 | 24.51824 | 23.72403 | 1.429 | 0.948 | 109.0 | 1.098 | 107.0 | 1.104 | 111.9 |  |
|  | *r_g_^VSCF^* | 125.30020 | 24.51924 | 23.70764 | 1.430 | 0.957 | 108.6 | 1.098 | 106.9 | 1.105 | 111.9 |  |
|  | *r_g_^VCI(5)^* | 125.93130 | 24.51465 | 23.71850 | 1.429 | 0.949 | 108.8 | 1.098 | 106.9 | 1.104 | 111.9 |  |
| **S4** | *r_e_^BO^* | 129.22905 | 24.89401 | 24.05655 | 1.418 | 0.956 | 109.8 | 1.089 | 109.8 | 1.089 | 109.8 |  |
|  | *r_g_^VSCF^(3D)* | 125.71747 | 24.60086 | 23.78708 | 1.426 | 0.956 | 108.8 | 1.097 | 106.9 | 1.103 | 111.9 |  |
|  | *r_g_^VCI(4)^* | 126.41959 | 24.59772 | 23.80254 | 1.435 | 0.951 | 108.8 | 1.100 | 106.9 | 1.106 | 111.8 |  |
|  | *r_g_^VSCF^* | 125.71780 | 24.59945 | 23.78669 | 1.426 | 0.956 | 108.8 | 1.097 | 106.9 | 1.103 | 111.9 |  |
|  | *r_g_^VCI(5)^* | 126.35293 | 24.59502 | 23.79783 | 1.426 | 0.948 | 109.0 | 1.096 | 107.0 | 1.102 | 111.9 |  |
| **S5** | *r_e_^BO^* | 128.75721 | 24.65181 | 23.82693 | 1.424 | 0.960 | 110.0 | 1.092 | 110.0 | 1.092 | 110.0 |  |
|  | *r_g_^VSCF^(3D)* | 125.52109 | 24.38272 | 23.58429 | 1.432 | 0.959 | 109.5 | 1.099 | 106.9 | 1.106 | 112.0 |  |
|  | *r_g_^VCI(4)^* | 126.33456 | 24.38317 | 23.60606 | 1.431 | 0.950 | 110.1 | 1.099 | 107.0 | 1.099 | 112.0 |  |
|  | *r_g_^VSCF^* | 125.51990 | 24.37868 | 23.58172 | 1.432 | 0.959 | 109.5 | 1.099 | 106.9 | 1.099 | 112.0 |  |
|  | *r_g_^VCI(5)^* | 126.19630 | 24.37353 | 23.59312 | 1.432 | 0.951 | 109.8 | 1.099 | 107.0 | 1.099 | 112.0 |  |
|  |  |  |  |  |  |  |  |  |  |  |  |  |
| Iijima88 [1] | ED & MW, *r_z_* | 126.37635 | 24.64783 | 23.63020 | 1.428 | 0.975 | 107.6 | 1.098 |  |  |  | 109.1 |
| Herbst84 [2] | MMW & THz,  r_effective_ | 127.63075 | 24.68418 | 23.76537 |  |  |  |  |  |  |  |  |
| Benston84 [3] | ED, *r_a_* |  |  |  | 1.428 | 0.960 |  | 1.096 | 106.8 | 1.102 | 112.6 |  |
| Gerry76 [4] | MW & MMW, *r_S_* | 127.57103 | 24.68020 | 23.76979 | 1.421 | 0.963 | 108.0 | 1.094 |  |  |  | 108.32 |
| Lees68 [5] | MMW, *r_s_* |  |  |  | 1.425 | 0.945 | 108.3 | 1.094 |  |  |  | 108.38 |
| Kimura59 [6] | ED |  |  |  | 1.428 | 0.960 | 109.0 | 1.095 |  |  |  | 109.28 |
| Nishikawa56 [7] | MW |  |  |  | 1.427 | 0.953 | 108.2 | 1.096 |  |  |  | 109.4 |
| Swalen55 [8] | MW |  |  |  | 1.428 | 0.967 | 107.2 | 1.098 |  |  |  | 109.6 |
| Venkateswarlu55 [9] | MW |  |  |  | 1.427 | 0.956 | 108.5 | 1.096 |  |  |  | 109.2 |
| Ivash53 [10] | MW |  |  |  | 1.434 | 0.937 | 105.6 | 1.093 |  |  |  | 109.3 |
| **Setup** | **Parameter** | **A / GHz** | **B / GHz** | **C / GHz** | **r_CO_ / Å** | **r_OH_ / Å** | **α_COH_ / °** | **r_CH_ / Å** | **α_OCH_ / °** | **r_CH,oop_ / Å** | **α_OCH,oop_ / °** | **α_HCH_ / °** |

**Structural parameters calculated for methanol with various setting**

Table S1 comprises structural parameters (rotational constants, bond lengths and angles). We distinguish between Born-Oppenheimer equilibrium parameters (r_e_) and vibrationally-averaged structural parameters (*r_g_*). The data used in the evaluation is highlighted blue.

**References**

1. Iijima T (1989) Zero-point average structure of methanol. J Mol Struct 212:137–141. https://doi.org/10.1016/0022-2860(89)85073-2

2. Herbst E, Messer JK, De Lucia FC, Helminger P (1984) A new analysis and additional measurements of the millimeter and submillimeter spectrum of methanol. J Mol Spectrosc 108:42–57. https://doi.org/10.1016/0022-2852(84)90285-6

3. Benston OJ, Ewbank JD, Paul DW, et al (1984) Multichannel Densitometry of Gas Electron Diffraction Patterns. Appl Spectrosc 38:204–208. https://doi.org/10.1366/0003702844554107

4. Gerry MCL, Lees RM, Winnewisser G (1976) The torsion-rotation microwave spectrum of 12CH318OH and the structure of methanol. J Mol Spectrosc 61:231–242. https://doi.org/10.1016/0022-2852(76)90245-9

5. Lees RM, Baker JG (1968) Torsion-vibration-rotation interactions in methanol. I. Millimeter wave spectrum. J Chem Phys 48:5299–5318. https://doi.org/10.1063/1.1668221

6. Kimura K, Kubo M (1959) Structures of Dimethyl Ether and Methyl Alcohol. J Chem Phys 30:151–158. https://doi.org/10.1063/1.1729867

7. Nishikawa T (1956) Fine Structure of J =1←0 Transition due to Internal Rotation in Methyl Alcohol. J Phys Soc Japan 11:781–786. https://doi.org/10.1143/JPSJ.11.781

8. Swalen JD (1955) Structure and potential barrier to hindered rotation in methyl alcohol. J Chem Phys 23:1739–1740. https://doi.org/10.1063/1.1742449

9. Venkateswarlu P, Gordy W (1955) Methyl Alcohol. II. Molecular Structure. J Chem Phys 23:1200–1202. https://doi.org/10.1063/1.1742240

10. Ivash E V., Dennison DM (1953) The methyl alcohol molecule and its microwave spectrum. J Chem Phys 21:1804–1816. https://doi.org/10.1063/1.1698668
